# Transcriptome Analysis Clarified Genes Involved in Betalain Biosynthesis in the Fruit of Red Pitayas (*Hylocereus costaricensis*)

**DOI:** 10.3390/molecules24030445

**Published:** 2019-01-27

**Authors:** Xingyuan Xi, Yuan Zong, Shiming Li, Dong Cao, Xuemei Sun, Baolong Liu

**Affiliations:** 1 Qinghai Province Key Laboratory of Crop Molecular Breeding, Northwest Institute of Plateau Biology, Chinese Academy of Sciences, Xining 810008, China; xixy@nwipb.cas.cn (X.X.); laughing1898@icloud.com (Y.Z.); lishiming@genomics.cn (S.L.); caodong@nwipb.cas.cn (D.C.); 2 State Key Laboratory of Plateau Ecology and Agriculture, Qinghai University, Xining 800010, China; 3 Key Laboratory of Adaptation and Evolution of Plateau Biota, Northwest Institute of Plateau Biology, Chinese Academy of Sciences, Xining 810008, China; 4 Qinghai Key Laboratory of Genetics and Physiology of Vegetables, Qinghai University, Xining 810008, China; 5 University of Chinese Academy of Sciences, Beijing 100049, China

**Keywords:** pitayas, red flesh, transcriptome analysis, betalain biosynthesis

## Abstract

The red flesh trait gives red pitayas more healthful components and a higher price, while the genetic mechanism behind this trait is unknown. In this manuscript, transcriptome analysis was employed to discover the genetic differences between white and red flesh in pitayas. A total of 27.99 Gb clean data were obtained for four samples. Unigenes, 79,049 in number, were generated with an average length of 1333 bp, and 52,618 Unigenes were annotated. Compared with white flesh, the expression of 10,215 Unigenes was up-regulated, and 4853 Unigenes were down-regulated in red flesh. The metabolic pathways accounted for 64.6% of all differentially expressed Unigenes in KEGG pathways. The group with high betalain content in red flesh and all structural genes, related to betalain biosynthesis, had a higher expression in red flesh than white flesh. The expression of the key gene, tyrosine hydroxylase *CYP76AD1*, was up-regulated 245.08 times, while 4,5-DOPA dioxygenase *DODA* was up-regulated 6.46 times. Moreover, the special isomers *CYP76AD1α* and *DODAα* were only expressed in red flesh. The competitive anthocyanin biosynthesis pathway had a lower expression in red flesh. Two MYB transcription factors were of the same branch as *BvMYB1*, regulating betalain biosynthesis in beet, and those transcription factors had expression differences in two kinds of pitayas, which indicated that they should be candidate genes controlling betalain accumulation in red pitayas. This research would benefit from identifying the major gene controlling red flesh trait and breed new cultivars with the red flesh trait. Future research should aim to prove the role of each candidate gene in betalain biosynthesis in red pitayas.

## 1. Introduction

Pitayas, commonly known as dragon fruit, belong to the genus *Hylocereus* of the order Caryophyllales. *Hylocereus* species originate from Latin America and are cultivated in tropical and subtropical regions worldwide [1,2]. There are several types of pitayas, such as *Hylocereus undatus*, *Hylocereus costaricensis*, and *Hylocereus megalanthus*. Among them, *H. undatus* and *H. costaricensis* are the most widely cultivated and have the same red fruit skin color but different flesh colors, white and red, respectively. Two betalain pigments, namely, the red-violet betacyanins and yellow-orange betaxanthins, contribute to the red flesh color [3]. *H. costaricensis* has a high Betalain content, but some agricultural traits are worse than those of *H. undatus*. For example, *H. costaricensis* is a self-pollination plant and has a lower setting percentage due to its higher stigma. Understanding the genetic mechanism of the red flesh trait will be beneficial for breeding new cultivars carrying both red flesh and better agricultural traits.

Betalains are water-soluble nitrogen-containing pigments that also contain betalamic acid as the chromophore [4]. Unlike the ubiquitous anthocyanin and carotenoid classes of pigments, betalains are fairly rare in nature and are restricted to a single plant order, namely, Caryophyllales [4]. Within Caryophyllales, betalains occur in a mutually exclusive fashion with anthocyanins, as no plant has been found to naturally produce both types of pigments [5,6]. Betalains assume many of the roles of anthocyanins [7]—betalains can endow a wide range of plant tissues, including leaves, stem, fruits, flowers, roots, and seeds, with red-violet and yellow-orange color [8]. It also plays an important role in the attraction of pollinators and frugivores for fertilization and seed dispersal [4]. Additionally, betalains are likely to participate in plant defense against various biotic and abiotic stresses [9,10,11,12,13,14,15,16,17,18]. The advantage of betalain color is that the color does not depend on pH and is more stable than that from anthocyanins, which is a natural colorant [7]. Furthermore, betalain pigments have been studied for their antioxidant and antiradical activities, and for the chemoprevention of cancer [19,20,21].

The betalain biosynthesis pathway is relative clear. First, tyrosine forms l-3,4-dihydroxyphenylalanine (l-DOPA) [22,23] are catalyzed by tyrosine hydroxylase CYP76AD1, CYP76AD5, or CYP76AD6, and l-DOPA forms 4,5-seco-DOPA catalyzed by 4,5-DOPA dioxygenase (DODA), which then produces betalamic acid [24]. l-DOPA can also produce DOPA-quinone under the oxidation of polyphenol oxidase -, and then shrink to form cyclo-DOPA [25], which is the universal substrate for producing betalains. Tyrosine hydroxylase CYP76AD1 is a bifunctional enzyme that also converts DOPA into cyclo-DOPA [26]. B5GT, B6GT, and cDOPA5GT are involved in the glycosylase reaction -. B5GT and B6GT catalyze betanidin to betanin [27,28], and cDOPA5GT catalyzes cyclo-DOPA glycosylation [29]. The formation of betaxanthin requires the spontaneous binding of betalamic acid and amino acid amines [30], while betacyanin is formed by the acetylation of betanin [31]. The formation of betanin generally requires cyclo-DOPA to bind with betalamic acid to form betanidin [31], then the betanidin glycosylateto forms betanin [31]. However, in some Caryophyllaceae plants, cyclo-DOPA-5-*O*-glucoside can also directly bind to betalamic acid to form betanin spontaneously [29,32]. The whole pathway is regulated by the MYB transcription factor BvMYB1 in beet [33].

High-throughput RNA sequencing (RNA-Seq) has emerged as a powerful and cost-efficient tool for studying transcript profiling and obtaining the nucleotide sequence of genes expressed in various plant species. In this article, RNA-Seq was employed to determine the transcriptome difference between the red and white flesh of pitayas, and to isolate the dominant genes related to betalain biosynthesis.

## 2. Results

### 2.1. Illumina Sequencing and Unigenes Assembly

The total raw reads were 186.66 M for four samples in transcriptome analysis, and a total of 27.99 Gb clean data were obtained after filtering. The filtered readings were over 99%, and both Q20 and Q30 were over 95%, which means the Illumina sequencing was of high quality for further analysis (Table S1). Trinity software was used for assembling Unigenes. A total of 79,049 Unigenes were generated, with an average length of 1333 bp and an N50 length of 2163 bp (Table S2). A total of 52,618 Unigenes were annotated in the database as Nr, Nt, Swissprot, KEGG, and KOG, accounting for 66.56% of the 79,049 Unigenes (Table 1).

### 2.2. Differentially Expressed Unigenes in Red and White Flesh

**Figure 1:**
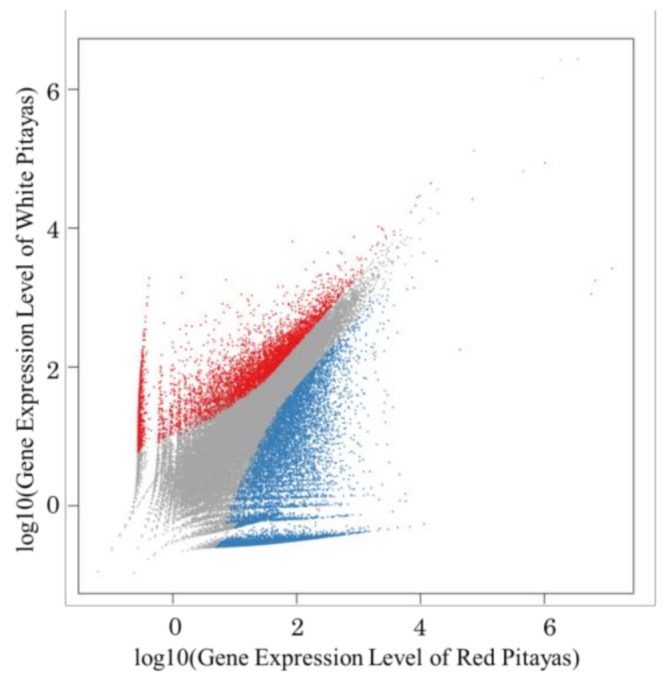
Differentially expressed genes between red pitayas and white pitayas. The genes were classified into three classes. Blue genes are up-regulated if the gene expression of the red pitayas is larger than that of the white pitayas. Red genes are down-regulated if the gene expression of the red pitayas is less than that of the white pitayas. Grey genes are not differentially expressed.

The 79,049 Unigene expression levels were calculated by the FPKM values (fragments per kb per million reads). Compared with white fruit, the expression of 10,215 Unigenes was up-regulated, and 4853 Unigenes were down-regulated in red fruits (Figure 1).

**Figure 2:**
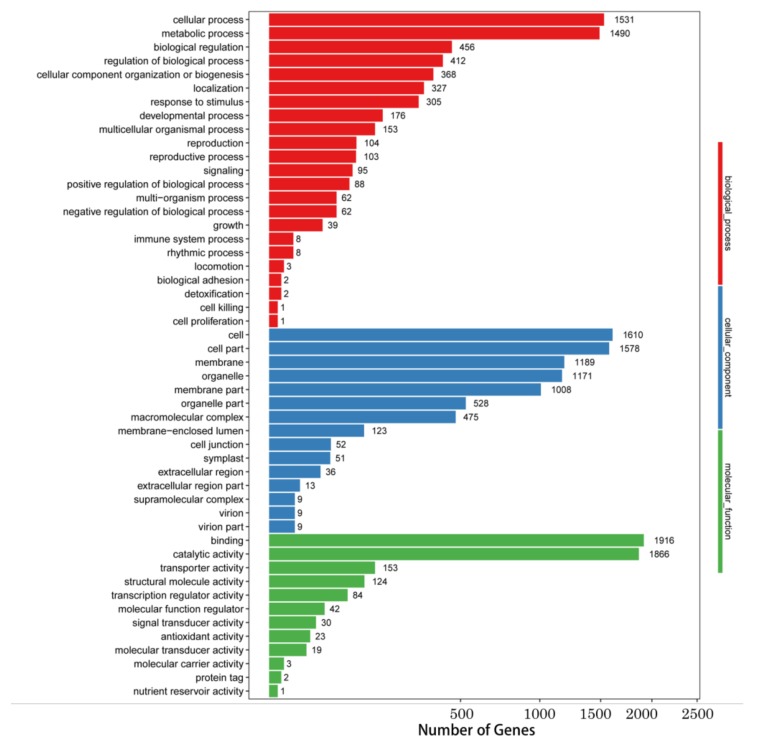
Gene ontology (GO) classifications of differentially expressed Unigenes. Unigenes were assigned to three categories: Biological processes, cellular components, and molecular functions.

Gene ontology (GO) functions analyzed 14,378 differentially expressed Unigenes, of which 5796 differentially expressed Unigenes were involved in biosynthetic pathways, 7861 Unigenes were related to cellular components, and 4253 Unigenes were related to molecular functions (Figure 2). In biosynthetic pathways, differentially expressed Unigenes were enriched in pathways related to cell metabolism and other metabolic pathways. Unigenes that were differentially expressed in relation to cell components are mainly concentrated in cells, cell membranes, and organelles. Most differentially expressed Unigenes were associated with binding and catalytic activity in the molecular functional classification.

**Figure 3:**
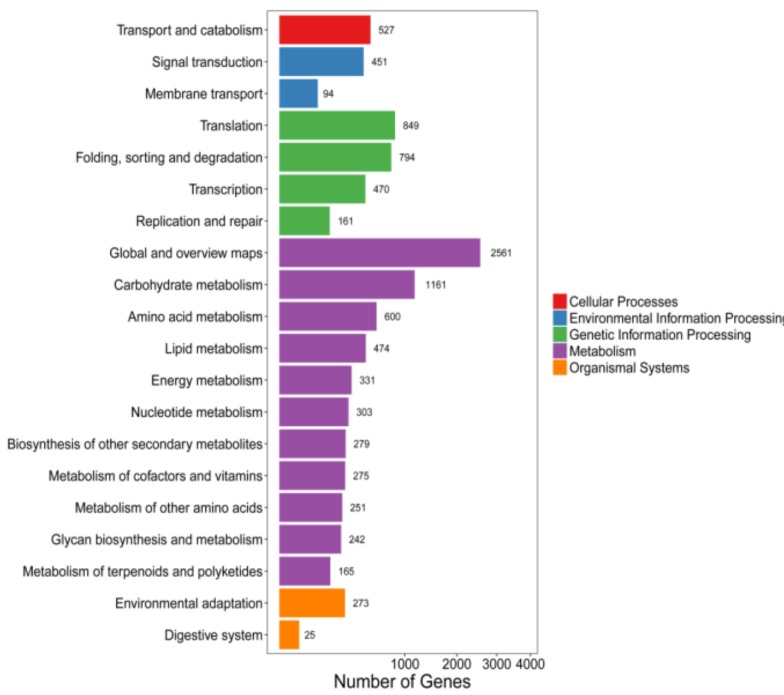
KEGG pathway of differentially expressed Unigenes. Unigenes were assigned to five categories: Cellular processes, environmental information processing, genetic information processing, metabolism, and organismal systems.

Unigenes, 38,851 in number, were annotated in the KEGG database, and only 10,286 Unigenes exhibited different expression levels. The metabolic pathways and genetic signaling pathways accounted for 64.6% and 22.1% of all differentially expressed Unigenes, respectively (Figure 3). The metabolic pathways were mainly concentrated in carbohydrate metabolism and amino acid metabolism. The enriched amino acid metabolism pathway included valine, leucine and isoleucine, glycine, serine, threonine, beta-alanine, and phenylpropanoid metabolism biosynthesis.

### 2.3. Unigenes in the Betalain Biosynthesis Pathway and Their Expression Character

**Figure 4:**
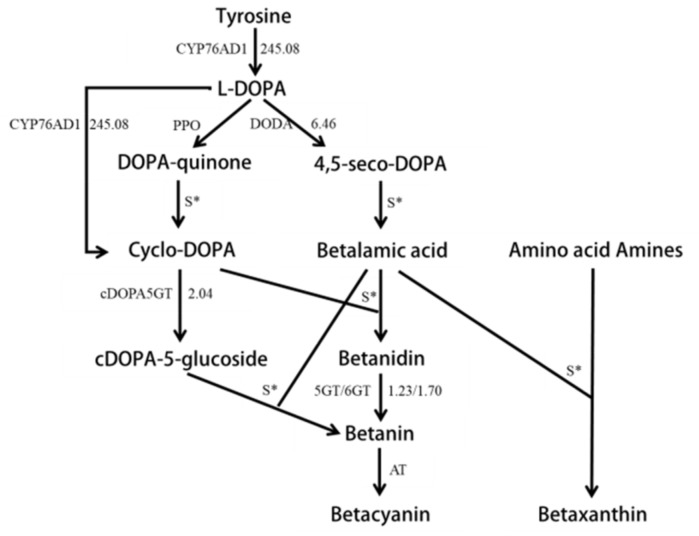
The differential expression of structural genes in the tyrosine pathway. Arrows show the metabolic stream, left or upward arrows represent the genes catalyzing the progress, and the number represents the up-regulation times of the genes in red pitayas compared to white pitayas.

The main pigments of red pitaya are betalains, based on chemical experiments [3]. The Unigenes related to betalain biosynthesis were selected through blastx software to compare their expression difference in different color fleshes. A total of 19 Unigenes were found to be homologous to the five betalain biosynthesis structural genes *CYP76AD1*, *DODA*, *CDOPA5GT*, *5GT,* and *6GT*. The sequence lengths of all different expressions of Unigenes were obviously longer than the reference sequences (Table S3). Nine Unigenes had higher transcript levels in red flesh than white flesh, while only one Unigene showed a higher transcript level in white flesh. Generally, all structural genes had a higher expression level in red pitaya than white pitaya based on the sum FPKM values of all homologous Unigenes. The expression of tyrosine hydroxylase CYP76AD1, a key enzyme in the first step of the betaine metabolism pathway, was up-regulated 245.08 times (Figure 4; Table S3). The two homologous Unigenes, Unigene14,941 and Unigene24,870, belonged to *CYP76AD1*-α, γ, respectively, based on Brockington’s classification system [34] (Figures S1 and S5). The expression level of Unigene14,941 in red flesh was 1231.105 times that of white flesh, while the expression level of Unigenes24,870 was not significant between red and white flesh (Table S3). Unigene14,941 should be the candidate gene that plays the role of *CYP76AD1* in betalain biosynthesis.

4,5-DOPA dioxygenase (DODA) was another key enzyme to catalyze dihydroxyphenylalanine (DOPA) to 4,5-seco-DOPA in the betalain biosynthesis pathway. Eight Unigenes were homologous to *DODA*, and six Unigenes were from the same contigs. The expression of *DODA* was up-regulated 6.46 times in red flesh as compared to white flesh, based on the sum FPKM values of all homologous Unigenes (Figure 4; Table S3). Each homologous Unigene was up-regulated from 0.506 to 485.939 times (Table S3). Furthermore, four Unigenes—Unigene3374, Unigene19,015, CL3967.Contig4, and CL3967.Contig6—were up-regulated over 100 times. *DODA* contained two subunit types [34], but all eight Unigenes belonged to *DODA-*α in this research (Figures S2 and S6). *DODA-*α could be classified into *DODA-*α*1* and *DODA-*α*2* [35]. *DODA1* had a function in the synthesis of betaine, while *DODA2* had no function due to the allelic variation of amino acids at seven sites [35]. Based on the seven loci differences, Unigene3374 and Unigene19,015 belonged to *DODA-*α*1*, while six CL3967 Unigenes were homologous to *DODA-*α*2*. Due to the early termination of Unigene19,015, Unigene3374 should be the candidate gene for playing the *DODA* function in the betalain synthesis pathway.

cDOPA5GT catalyzed the glycosylation of the cDOPA 5′O site to cDOPA-5-gluside in the betalain synthesis pathway. The expression level was up-regulated, on average, 2.04 times (Figure 4; Table S3). Three Unigenes were found to be homologous to *cDOPA5GT* (Figure S3), but only one Unigene—CL5813.Contig1—had a significantly expressed change in red flesh comparing with white flesh. The expression level was up-regulated 24.927 times (Table S3).

Both 5GT and 6GT can catalyzed betaine to produce betaine. Two and four Unigenes were homologous to 5GT and 6GT, respectively (Figure S4; Table S3). The Unigenes CL5200.Contig2 (5GT) and CL652.Contig2 (6GT) had significantly higher expression in red flesh than white flesh (Table S3). The relative expression levels of *5GT* and *6GT* were up-regulated in red flesh as compared to white flesh by an average of 1.226 and 1.698 times, respectively (Figure 4; Table S3).

### 2.4. The Expression Character of the Unigenes Relative to Anthocyanin Biosynthesis

**Figure 5:**
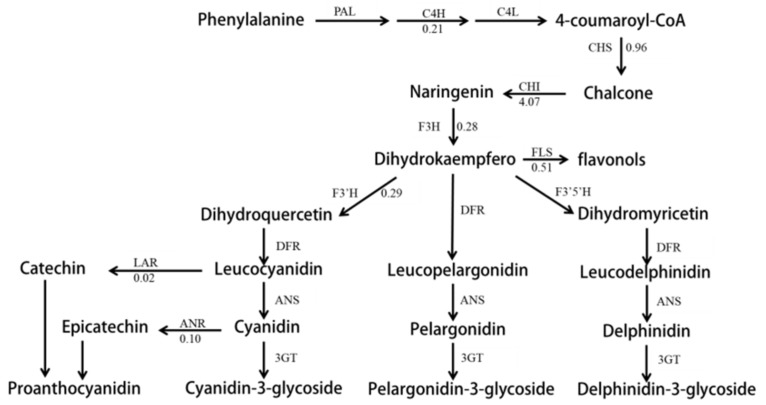
The differential expression of structural genes in the phenylalanine pathway. Arrows show the metabolic stream, left or upward arrows represent the genes catalyzing the progress, and the number represents the up-/down-regulation times of the genes in red pitayas as compared to white pitayas.

Betalains occurred in a mutually exclusive fashion with anthocyanins within the order Caryophyllales, and the betalain biosynthesis pathway was up-regulated in red pitayas. To evaluate the effect of betalain biosynthesis on anthocyanin biosynthesis, the expression level of Unigenes relative to the anthocyanin biosynthesis pathway was compared in red and white pitaya flesh. Eleven structure genes were used as a reference sequence to find homologous Unigenes, while only eight structural genes had the homologous sequence. Flavonoid-3′,5′-hydroxylase (F3′5′H), dihydroxyflavonol reductase (DFR), and anthocyanin synthase (ANS) were not detected in the Unigene database, while all homologous sequences were shorter than the reference sequence for eight structural genes. The expression levels of cinnamic acid 4-hydroxylase (C4H), chalcone synthase (CHS), flavonol-3-hydroxylase (F3H), flavonoid-3′-hydroxylase (F3′H), anthocyanin reductase (LAR), anthocyanin reductase (ANR), and flavonol synthase (FLS) in red flesh were 0.21, 0.96, 0.28, 0.29, 0.02, 0.10, and 0.51 times less than that of white flesh, respectively (Figure 5; Table S3). Only chalcone isomerase (CHI) had a higher expression level in red flesh.

### 2.5. The Key MYB Regulation Factor Regulating Betalain Biosynthesis

**Figure 6:**
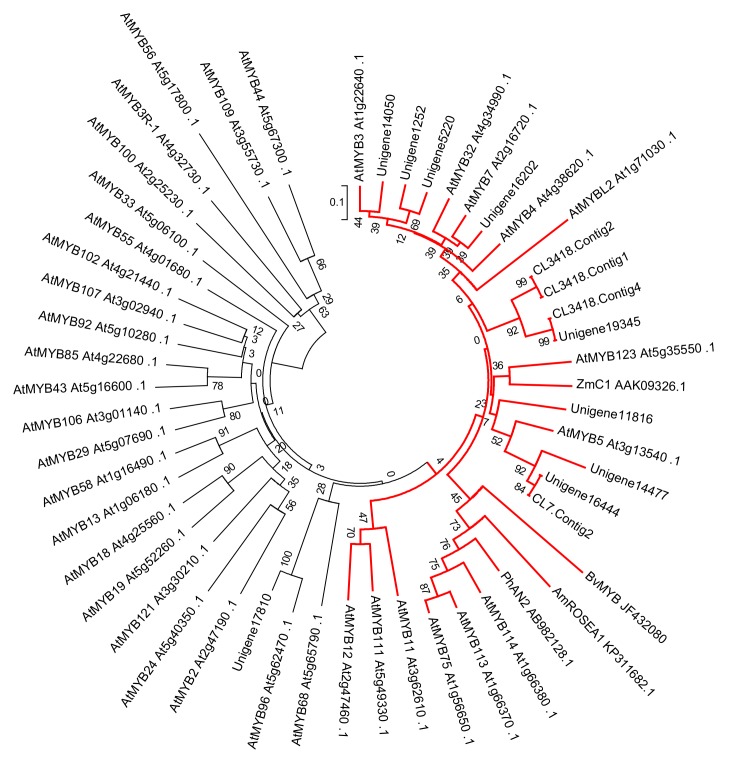
Maximum likelihood tree of MYBs in pitayas and R2R3 MYBs in *Arabidopsis thaliana* constructed by MEGA6 using the resources at IT3F (An Interspecies Transcription Factor Function Finder for Plants). Unigene11,816, CL7.contig2, Unigene16,444, Unigene14,477, CL3418.Contig1, CL3418.Contig2, CL3418.Contig4, Unigene19,345, Unigene16,202, Unigene15,050, Unigene5220, and Unigene1252 form a monophyletic clade with MYB transcriptors controlling anthocyanins and proanthocyanins biosynthesis, as depicted by red lines.

*BvMYB1* was the first isolated transcription factor regulating betalain biosynthesis in beet. Interestingly, *BvMYB1* belonged to the same branch of the MYB transcriptor factors regulating anthocyanin biosynthesis in phylogenetic trees [33]. In this research, BvMYB1 protein was used as a reference sequence for blastx analysis to identify the candidate gene controlling betalain biosynthesis. Thirteen MYB transcriptor Unigenes were selected with scores over 130 to construct the phylogenetic tree containing *BvMYB1* and the R2R3 MYB transcriptors from *Arabidopsis*. Twelve Unigenes belonged to the branch regulating the phenylalanine metabolic pathway in phylogenetic tress, while one (Unigene17,810) was close to *AtMYB96*, which mediates abscisic acid signaling during the drought stress response and pathogen resistance response in *Arabidopsis* [36,37] (Figure 6), which in turn implies that the MYBs in the phenylalanine metabolic pathway had been isolated from the RNA-Seq database. Only CL3418.Contig2 and CL7.Contig2 had a higher transcript level in red flesh than white flesh, as they were up-regulated by 227.725 and 444.872 times in all of the 12 Unigenes (Table S3). CL3418.Contig2 and CL7.Contig2 belonged to the branch of phenylalanine biosynthesis, and had the same expression profile as *BvMYB1*, which indicated that the two Unigenes were the candidate genes for betalain biosynthesis.

## 3. Discussion

RNA-Seq has been proven to be a good way to isolate the key genes for anthocyanin biosynthesis in tissue with a high anthocyanin content. It could also be used here to obtain the genes related to betalain biosynthesis in pitayas without genome sequence information. One Unigene of each structural gene in the betalain biosynthesis pathway and two Unigenes of the regulated genes were discovered in this manuscript. The whole coding sequences of Unigenes can be found in the current sequencing depth. More work should be undertaken to prove the real function of each candidate gene in betalain biosynthesis. Plant color was usually proven by the betalains, anthocyanin, carotenoid, and chlorophyⅡ [4]. Betalain and anthocyanin can provide the plant with red color [4]. In red pitayas, all structural genes in anthocyanin biosynthesis could not be found using the whole coding sequence, which means that the expression level of anthocyanin biosynthesis was lower than that of betalain biosynthesis. Moreover, the key structural genes *F3′5′H*, *DFR*, and *ANS* could not be detected in the Unigenes database. Compared with white pitayas, all structural genes of betalain biosynthesis had higher expression levels in red pitayas. These results confirm that the betalain causes red flesh in pitayas, not anthocyanin.

Betalain synthesis in beet was regulated by *BvMYB1*, which is a transcription factor of the MYB class related to anthocyanin regulation. Silencing *BvMYB1* would down-regulate structural genes related to betaine synthesis, and overexpression of *BvMYB1* would up-regulate these genes [32]. In our research, the structural genes had a higher expression in red pitayas than white pitayas, which is the same as the phenotype of the beet Y locus *BvMYB1*. Two candidate MYB transcription factors could be obtained in response to the action of the betalain synthesis pathway in our research. The two MYB transcription factors were close to the genes regulating tannin and procyanidins biosynthesis in *Arabidopsis* [38], respectively. Both tannin and procyanidins biosynthesis belong to the phenylpropanoid biosynthesis pathway. The transcription factors, inducing some special compound accumulation in some species, could induce new compounds in other species. For example, *AtTT2* can promote procyanidin accumulation in the testa of *Arabidopsis* [39], while the homologous gene *ZmC1* could regulate anthocyanin biosynthesis in maize [40]. The betalain biosynthesis pathway was derived from tyrosine biosynthesis, while anthocyanin biosynthesis belonged to the phenylalanine biosynthesis pathway. Both tyrosine biosynthesis and phenylalanine biosynthesis were derived from shikimate biosynthesis. Anthocyanin biosynthesis should compete with the same substrate as betalain biosynthesis, which could be the reason why anthocyanin and betalain never coexist in some species. It could also explain the MYB transcription factor and regulate anthocyanin and betalain biosynthesis, which belong to the same branch in phylogenetic tree.

## 4. Materials and Methods

### 4.1. Plant Materials, cDNA Preparation, and Illumina Sequencing

*Hylocereus costaricensis* and *Hylocereus undatus* were collected from the Jingdong Company (Beijing, China). Two different parts closest to the center of each pitayas flesh was collected for the total RNA extraction. The total RNA was extracted using the Tiangen RNAprep Pure Plant Kit (Tiangen, China) according to the standard protocol. The quality of the total RNA was checked by electrophoresis in a 1.0% agarose gel and the concentration of the total RNA was determined by NanoDrop (Thermo Scientific, Wilmington, DE, USA). The cDNA libraries were prepared according to the manufacturer’s instructions for mRNA-Seq sample preparation (Illumina Inc, San Diego, CA, USA). The cDNA library products were sequenced by Illumina paired-end sequencing technology with read lengths of 100 bp, and they were sequenced on an Illumina HiSeq 2000 instrument by Huada Technologies Co., Ltd. (Beijing, China).

### 4.2. Sequence Data Filtering and De Novo Assembly

Before assembly, the raw paired-end reads, produced from sequencing machines, were filtered to obtain high-quality clean reads. Low quality sequences were removed, including sequences with ambiguous bases (denoted with >5% ‘N’ in the sequence trace), low quality reads (the rate of reads with a quality value ≤10 was more than 20%), and reads with adaptors. After purity filtering was completed, the high-quality reads were assembled by Trinity with default parameters to construct unique consensus sequences [41].

### 4.3. Analysis of Differential Gene Expression

The Unigene expression levels were calculated by the FPKM values (fragments per kb per million reads). Unigenes that were differentially expressed between *Hylocereus costaricensis* and *Hylocereus undatus* were analyzed by the Chi-square test using IDEG6 software [42]. The false discovery rate (FDR) method was introduced to determine the threshold *p*-value at FDR ≤ 0.001; the absolute value of |log2Ratio| ≥ 1 was used as the threshold to determine the significance of the differential expression of Unigenes.

### 4.4. Gene Annotation and Classification

In order to perform functional annotation, the assembled Unigenes were submitted to a public database and compared with the NCBI non-redundant protein database (Nr), NCBI nucleotide sequence database (Nt), Swiss-Prot (http://www.uniprot.org/) [43]—Kyoto Encyclopedia of Genes and Genomes (KEGG) databases (http://www.genome.jp/kegg/) [44]—and KOG (ftp://ftp.ncbi.nih.gov/pub/COG/KOG) [45] using blastx (v.2.2.26) [46] with an e-value < 1 × 10^−5^. The blastx results were also used to train ESTScan [47].

The gene ontology (GO) [48] annotations were analyzed using the Blast2GO (V.2.5) program (http://www.geneontology.org). All differentially abundant Unigenes between the red and white flesh of cultivars *Hylocereus costaricensis* and *Hylocereus undatus* were mapped to the GO and KEGG pathway databases, and then the respective numbers of Unigenes for each GO and KEGG orthology (KO) term were calculated. To compare these Unigenes with the whole transcriptome background from pitayas, significantly enriched GO and KO terms from the set of differentially abundant Unigenes were identified using the hypergeometric test [49].

## 5. Conclusions

The red flesh trait gives red pitayas more healthful components and a higher price; however, the genetic mechanism behind this trait was previously unknown. In this study, transcriptome analysis was employed to discover the genetic differences between white and red flesh in pitayas. Here, a total of about 27.99 Gb clean RNA-Seq data were generated and de novo assembled into 79,049 Unigenes, in which 52,618 were annotated. The group with a high betalain content in red flesh and all structural genes related to betalain biosynthesis had a higher expression in red flesh than white flesh. The betalain-related genes were identified and characterized in the flesh of pitaya. One Unigene of each structural gene in the betalain biosynthesis pathway was discovered in this study. Unlike betainin synthesis, the expression of structural genes associated with anthocyanin synthesis were either reduced or undetectable. A previous study proved that anthocyanin MYB-like transcription factors activated betalain biosynthesis [33]. This indicated that MYB transcription factors associating with anthocyanin biosynthesis also could regulate betalain accumulation in red pitayas. Thus, we found that two MYB genes might regulate betalain accumulation. Further work should be conducted to strengthen the case for proving the real function of each candidate gene in betalain biosynthesis.

## Figures and Tables

**Table 1:** The annotation of assembled Unigenes.

Values	Total	Nr ^a^	Nt ^b^	Swissprot ^c^	KEGG ^d^	KOG ^e^	GO ^f^	Overall
Number	79,049	51,093	39,651	36,059	38,851	39,492	14,378	52,618
Percentage	100%	64.63%	50.16%	45.62%	49.15%	49.96%	18.19%	66.56%

^a^ NCBI non-redundant protein database; ^b^ NCBI nucleotide sequence database; ^c^ UniProt: A hub for protein information; ^d^ Kyoto Encyclopedia of Genes and Genomes database; ^e^ Clusters of Orthologous Groups database^; f^ Gene Ontology database.

## Supplementary Materials

The following are available online at http://www.mdpi.com/1420-3049/24/3/445/s1, Figure S1: Phylogeny of the CYP76AD1 lineage, Figure S2: Phylogeny of the DODA lineage, Figure S3: Phylogeny of the cDOPA5GT lineage, Figure S4: Phylogeny of the 5GT and 6GT lineage Figure S5: Amino acid sequences alignment of CYP76AD1, Figure S6: Amino acid sequences alignment of DODA. Table S1. Summary of transcriptome sequencing data in *Hylocereus undatus* and *Hylocereus costaricensis*, Table S2. Summary statistics of functional annotation for unigenes, Table S3. Differential expression analysis of Unigenes related to betalains and anthocyanins biosynthesis.
