# Supplementary material for: Transcriptome Analysis Clarified Genes Involved in Betalain Biosynthesis in the Fruit of Red Pitayas (Hylocereus costaricensis)
Source: Molecules. 2019 Jan 27;24(3):445. doi: 10.3390/molecules24030445 (PMC6384678; doi:10.3390/molecules24030445)
Supplement: Supplementary file 1 [file molecules-24-00445-s001.pdf]

*Article*

# Transcriptome analysis discovered the secrets of betalain biosynthesis in the fruit of Red Pitayas (*Hylocereus costaricensis*)

**Xingyuan Xi1+, Yuan Zong1,2+, Shiming Li1,3, Dong Cao1,2,3, Xuemei Sun4,5*, Baolong Liu1,2,3,5***

[1] Qinghai Province Key Laboratory of Crop Molecular Breeding, Northwest Institute of Plateau Biology, Chinese Academy of Sciences, Xining, Qinghai 810008, China.
[2] State Key Laboratory of Plateau Ecology and Agriculture, Qinghai University, Xining, Qinghai 800010, China.
[3] Key Laboratory of Adaptation and Evolution of Plateau Biota, Northwest Institute of Plateau Biology, Chinese Academy of Sciences, Xining, Qinghai 810008, China.
[4] Qinghai Key Laboratory of Genetics and Physiology of Vegetables, Qinghai University, Xining, Qinghai 810008, China.
[5] University of Chinese Academy of Sciences, Beijing 100049, China
[+]These authors contributed equally to this work.
*Corresponding author: 21875800@qq.com, blliu@nwipb.cas.cn

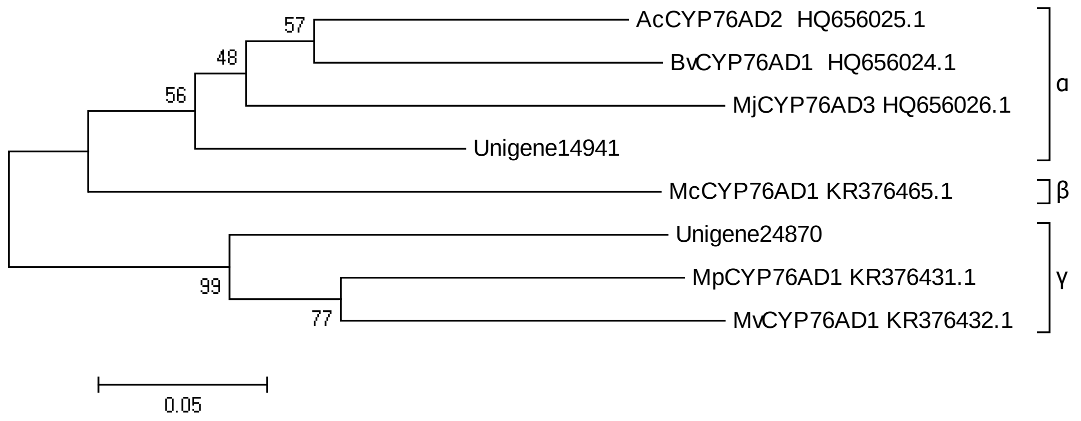


**Figure S1.** Phylogeny of the CYP76AD1 lineage, delimiting the CYP76AD1-$\alpha$, -β and –$\gamma$ clades. CYP76AD1 contained three subunit types, CYP76AD1-$\alpha$ recovered in *Amaranthus cruentus*, *Beta vulgaris* and *Mirabilis jalapa*, respectively. CYP76AD1-β recovered in *Mollugo cerviana*; CYP76AD1-$\gamma$ recovered in *Mollugo pentaphylla* and *Mollugo verticillata* [34]. Unigene 14941 form a clade with CYP76AD1-$\alpha$ and Unigene24870 form a clade with CYP76AD1-$\gamma$.

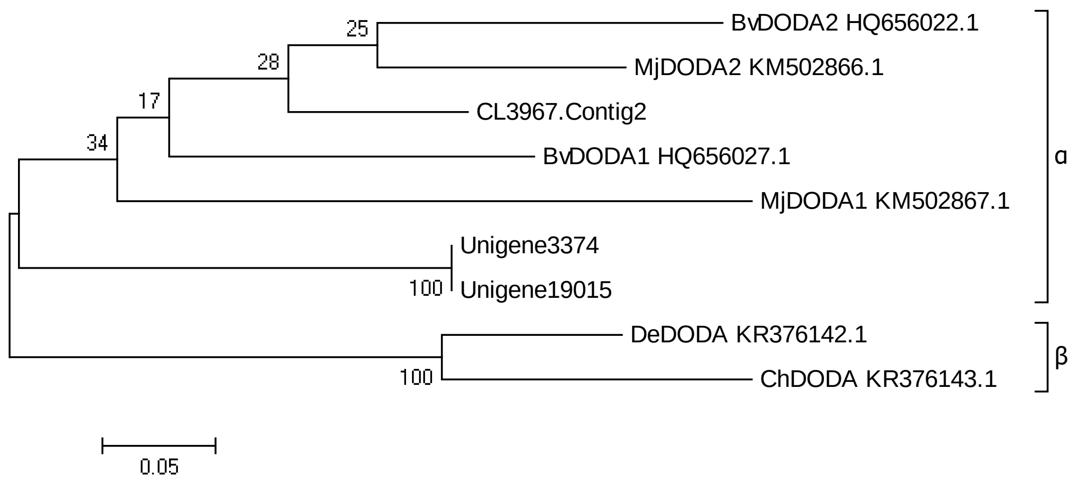


**Figure S2.** Phylogeny of the DODA lineage, delimiting the DODA-$\alpha$, -β and –$\gamma$ clades. DODA contained two subunit types, DODA–$\alpha$ recovered in *Beta vulgaris* and *Mirabilis jalapa*. DODA–β recovered in *Delosperma echinatum* and *Cypselea humifusa* [34]. CL3967, Unigene3374 and Unigene19015 form a clade with DODA –$\alpha$.

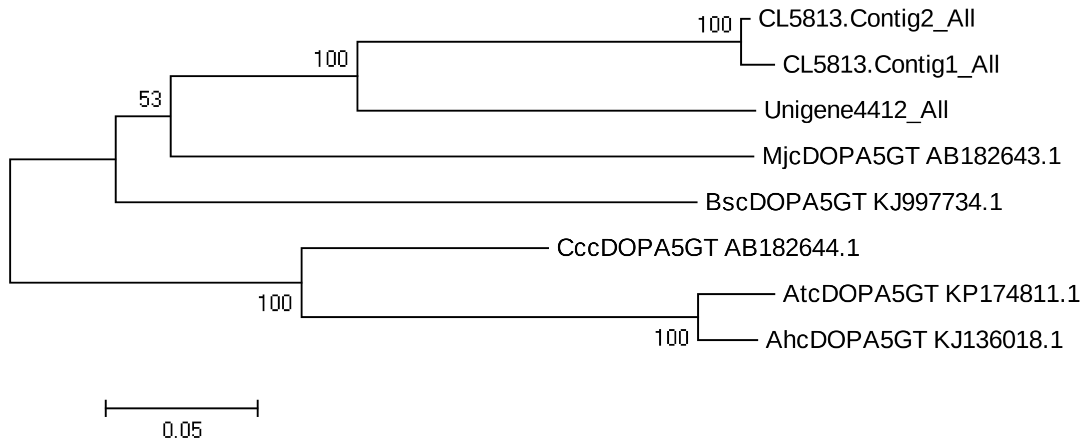


**Figure S3.** Phylogeny of the cDOPA5GT lineage. CL5813.Contig1, CL5813.Contig1 and Unigene4412 form a clade with cDOPA5GT in variety of species, which include *Mirabilis jalapa*, *Bougainvillea spectabilis*, *Celosia cristata*, *Amaranthus tricolor* and *Amaranthus hypochondriacus*.

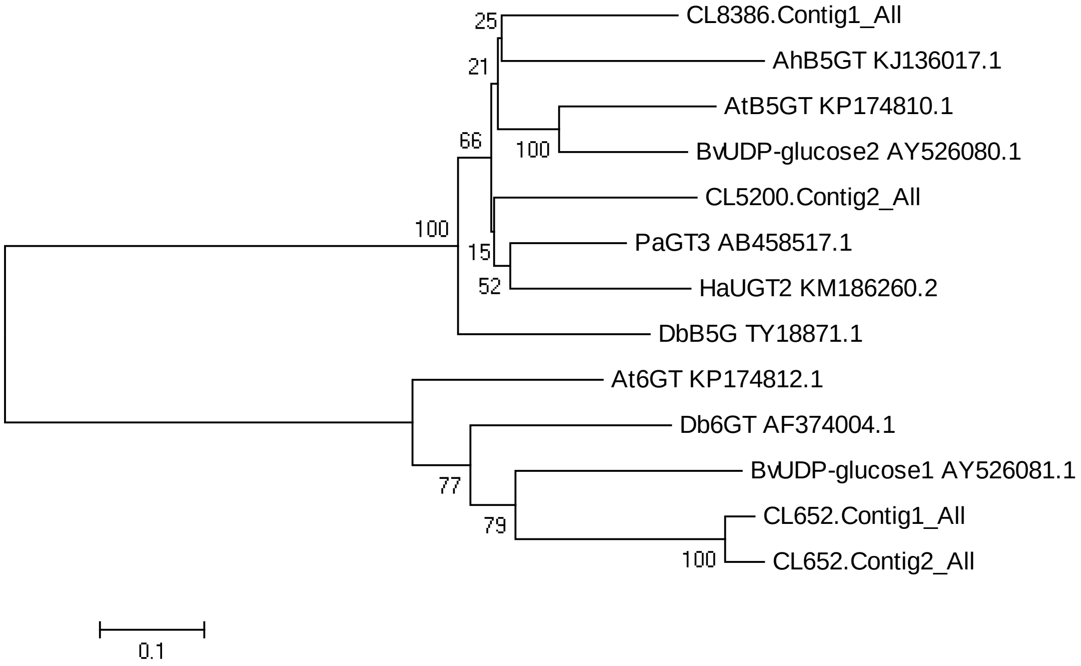


**Figure S4.** Phylogeny of the 5GT and 6GT lineage. CL5200.Contig2 and CL8386.Contig1 form a clade with 5GT in *Amaranthus hypochondriacus, Amaranthus tricolor, Beta vulgaris ,Phytolacca Americana, Haloloxylon ammodedron, Dorotheanthus bellidiformis*. While CL652.Contig1 and CL652.Contig2 form a clade with 6GT in *Amaranthus tricolor, Dorotheanthus bellidiformis, Beta vulgaris*.

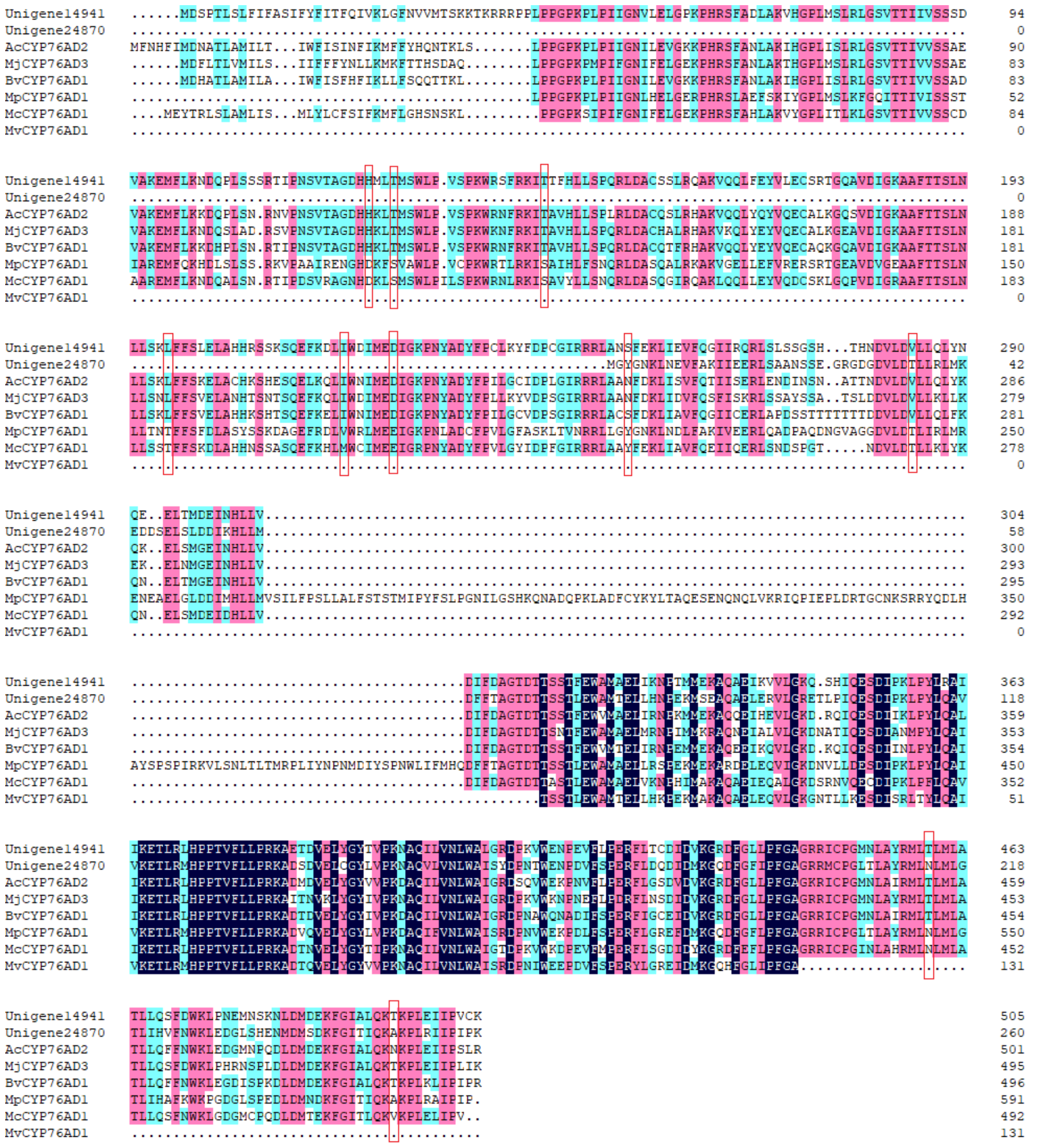

**Figure S5.** Amino acid sequences alignment of CYP76AD1, red in-line indicates the isomer-invariant residue. Based on Brockington's classification system, for CYP76AD1, several residues were also found to be diagnostic for the CYP76AD1-α clade, including histidine at site 111, threonine at sites 114 and 131, leucine at site 186, isoleucine at site 207, aspartic acid at site 213, asparagine at site 241 and valine at site 275, threonine at site 450, threonine at the site 486. While several residues were found to be diagnostic for the CYP76AD1-β clade, including aspartic acid at site 111, serine at sites 114 and 131, threonine at site 186, methionine at site 207, glutamic acid at site 213, tyrosine at site 241 and threonine at site 275, asparagine at site 450, valine at the site 486 [34]. CYP76AD1-β is closest to CYP76AD1-γ. Several residues were found to be diagnostic for the CYP76AD1-γ clade, including aspartic acid at site 111, serine at sites 114 and 131, threonine at site 186, glutamic acid at site 213, tyrosine at site 241 and threonine at site 275, asparagine at site 450, valine at the site 486. Residues are numbered on the basis of B. vulgaris CYP76AD1-α. Unigene 14941 is closest to CYP76AD1-α and Unigene24870 is closest to CYP76AD1-γ

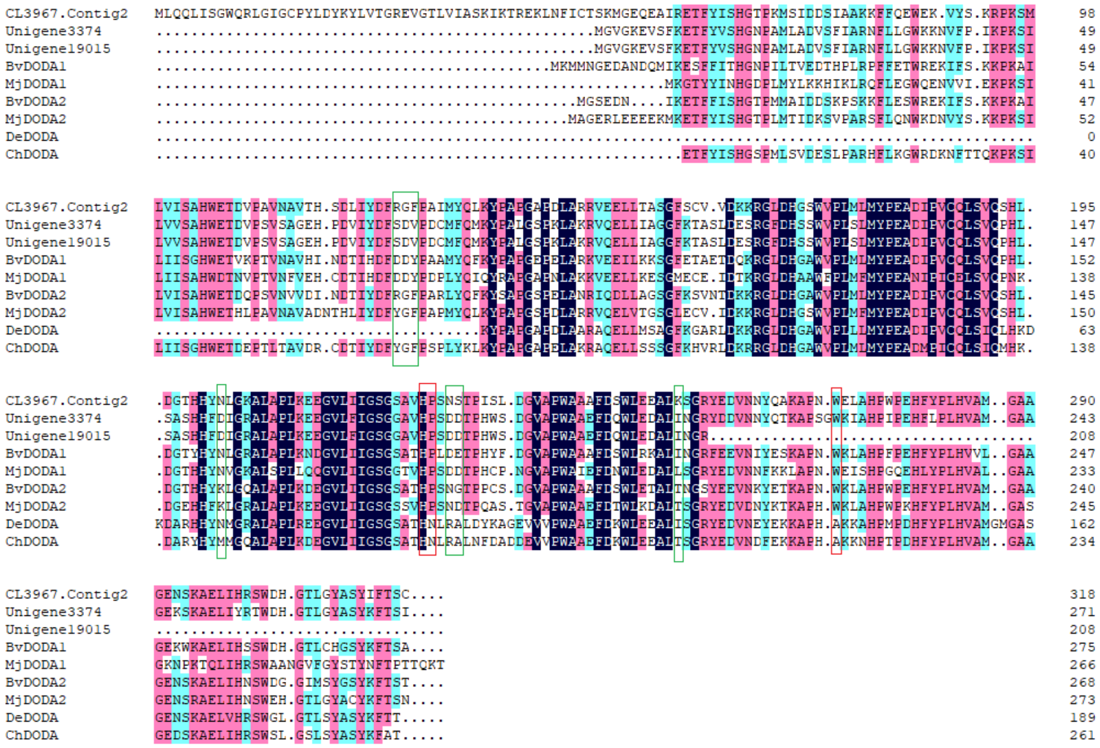

**Figure S6.** Amino acid sequences alignment of DODA, the red box line indicates the conserved residues of each isomer, and the green box line indicates the conserved residues to classify DODA1 and DODA2. Based on Brockington's classification system, two residues were diagnostic for the DODA-a clade, including proline at site 176 and asparagine at site 220. Two residues were diagnostic for the DODA-a clade, including proline at site 176 and asparagine at site 220. Two residues are diagnostic for the DODA-β clade, including asparagine at site 176 and alanine at site 220 [34]. CL3967, Unigene3374 and Unigene19015 are closest to DODA –α. Seven residues that are diagnostic for the DODA1 clade, including aspartic acid, aspartic acid and Tyrosine at site 74, 75 and 76, respectively, Asparagine at site 152, aspartic acid at site 178, glutamic acid at site 179, Isoleucine at site 203 [35]. CL3967 belongs to DODA2, while Unigene3374 and Unigene19015 are DODA1-like. Although Unigene19015 and Unigene3374 have the same amino acid sequences, Unigene19015 has the early termination at site 208. Residues are numbered on the basis of *B. vulgaris* DODA2.

**Table S1.** Summary of transcriptome sequencing data in *Hylocereus undatus* and *Hylocereus costaricensis*.

| Sample | Total Raw Reads(M) | Total Clean Reads(M) | Total Clean Bases (Gb) | Clean Reads Q20 (%) | Clean Reads Q30 (%) | Clean Reads Ratio(%) |
|---|---|---|---|---|---|---|
| red1 | 52.87 | 52.84 | 7.93 | 98.55 | 95.76 | 99.95 |
| red2 | 52.74 | 52.73 | 7.91 | 98.56 | 95.79 | 99.97 |
| white1 | 36.38 | 36.36 | 5.45 | 98.43 | 95.55 | 99.95 |
| white3 | 44.67 | 44.64 | 6.7 | 98.26 | 95.26 | 99.92 |

**Table S2.** Summary statistics of functional annotation for unigenes.

| Sample | Total Number | Total Length | Mean Length | N50 | N70 | N90 | GC(%) |
|---|---|---|---|---|---|---|---|
| red1 | 35203 | 32005035 | 909 | 1459 | 881 | 379 | 43.67 |
| red2 | 39033 | 38336515 | 982 | 1592 | 993 | 410 | 43.52 |
| white1 | 44167 | 51429992 | 1164 | 1856 | 1238 | 519 | 43.09 |
| white3 | 58225 | 71951833 | 1235 | 1945 | 1341 | 572 | 42.71 |
| All-Unigene | 79049 | 1.05E+08 | 1333 | 2163 | 1482 | 620 | 42.55 |

**Table S3.** Differential expression analysis of Unigenes related to betalains and anthocyanins biosynthesis.

| Pathway | Gene | KEGG ID (EC: NO) | Reference genes Length (bp) | GeneID | Length (bp) | Red-Expression | White-Expression | RED/WHITE | Up/Down-Regulation | Padj | Pvalue |
|---|---|---|---|---|---|---|---|---|---|---|---|
| Betalain | P450-CYP76AD | - | 1488 | Unigene14941 | 2129 | 2473.5980 | 2.0093 | 1231.1048 | Up | 0.0000 | 0.0000 |
| | | | | Unigene24870 | 1123 | 5.3334 | 8.1054 | 0.6580 | Down | 0.7751 | 0.5993 |
| | | | | Sum | | 2478.9314 | 10.1146 | 245.0834 | Up | | |
| | DODA | K15777 (1.13.11.-) | 810 | Unigene3374 | 1284 | 880.8943 | 1.7528 | 502.5507 | Up | 0.0000 | 0.0000 |
| | | | | Unigene19015 | 1592 | 39.1489 | 0.3090 | 126.6955 | Up | 0.0000 | 0.0000 |
| | | | | CL3967.Contig1 | 1533 | 25.4439 | 18.6377 | 1.3652 | Up | 0.7004 | 0.4929 |
| | | | | CL3967.Contig2 | 2037 | 215.9282 | 16.8624 | 12.8053 | Up | 0.0000 | 0.0000 |
| | | | | CL3967.Contig3 | 1549 | 85.2638 | 168.6405 | 0.5056 | Down | 0.0028 | 0.0004 |
| | | | | CL3967.Contig4 | 2429 | 164.5340 | 0.3386 | 485.9392 | Up | 0.0000 | 0.0000 |
| | | | | CL3967.Contig5 | 2933 | 22.3344 | 32.8289 | 0.6803 | Down | 0.5632 | 0.2873 |
| | | | | CL3967.Contig6 | 1471 | 115.9562 | 0.3205 | 361.7658 | Up | 0.0000 | 0.0000 |
| | | | | Sum | | 1549.5036 | 239.6904 | 6.4646 | Up | | |
| | cDOPA5GT | K15776 (2.4.1.-) | 1500 | CL5813.Contig1 | 1725 | 122.4929 | 4.9142 | 24.9263 | Up | 0.0000 | 0.0000 |
| | | | | CL5813.Contig2 | 2147 | 47.0533 | 80.1905 | 0.5868 | Down | 0.1166 | 0.0338 |
| | | | | Unigene4412 | 1420 | 36.8831 | 15.8661 | 2.3246 | Up | 0.1255 | 0.0370 |
| | | | | Sum | | 206.4292 | 100.9707 | 2.0444 | Up | | |
| | 5GT | K15774 | 1467 | CL8386.Contig1 | 1740 | 76.8512 | 105.1601 | 0.7308 | Down | 0.6697 | 0.4178 |

| | | | | | | | | | | | |
|---|---|---|---|---|---|---|---|---|---|---|---|
| | | (2.4.1.-) | | CL5200.Contig2 | 1449 | 58.4911 | 5.2783 | 11.0814 | Up | 0.0005 | 0.0001 |
| | | | | Sum | | 135.3423 | 110.4384 | 1.2255 | Up | | |
| | 6GT | K15775 (2.4.1.-) | 1443 | CL652.Contig1 | 1556 | 21.3411 | 82.7718 | 0.2578 | Down | 0.0082 | 0.0014 |
| | | | | CL652.Contig2 | 1717 | 124.7915 | 7.2972 | 17.1013 | Up | 0.0000 | 0.0000 |
| | | | | CL652.Contig3 | 482 | 2.7056 | 0.7965 | 3.3969 | Up | 0.6264 | 0.3450 |
| | | | | CL652.Contig4 | 518 | 6.3597 | 0.5264 | 12.0815 | Up | 0.1567 | 0.0489 |
| | | | | Sum | | 155.1979 | 91.3919 | 1.6982 | Up | | |
| Anthocyanin | C4H | K00487 (1.14.13.11) | 1620 | CL5457.Contig1 | 2030 | 426.3982 | 2021.5383 | 0.2109 | Down | 0.0000 | 0.0000 |
| | | | | Sum | | 426.3982 | 2021.5383 | 0.2109 | Down | | |
| | CHS | K00660 (2.3.1.74) | 669 | CL2124.Contig1 | 887 | 37.7740 | 1.9125 | 19.7511 | Up | 0.0000 | 0.0000 |
| | | | | CL2124.Contig2 | 685 | 0.6861 | 17.9800 | 0.0382 | Down | 0.0028 | 0.0004 |
| | | | | CL2124.Contig3 | 5300 | 72.6080 | 135.0453 | 0.5377 | Down | 0.2138 | 0.0733 |
| | | | | Unigene4340 | 2166 | 66.9432 | 30.6317 | 2.1854 | Up | 0.0772 | 0.0204 |
| | | | | Unigene6998 | 477 | 2.6340 | 3.1328 | 0.8408 | Down | 0.9191 | 0.8421 |
| | | | | Sum | | 180.6453 | 188.7024 | 0.9573 | Down | | |
| | CHI | K01859 (5.5.1.6) | 702 | Unigene36137 | 871 | 10.4987 | 0.3050 | 34.4220 | Up | 0.0108 | 0.0020 |
| | | | | Unigene6320 | 1151 | 40.9843 | 12.3565 | 3.3168 | Up | 0.0166 | 0.0033 |
| | | | | Sum | | 51.4830 | 12.6615 | 4.0661 | Up | | |
| | F3H | K00475 (1.14.11.9) | 1131 | Unigene4833 | 1587 | 73.6927 | 261.8570 | 0.2814 | Down | 0.0000 | 0.0000 |
| | | | | Sum | | 73.6927 | 261.8570 | 0.2814 | Down | | |
| | F3'H | K05280 (1.14.13.21) | 1608 | Unigene14914 | 1721 | 9.8985 | 34.6906 | 0.2853 | Down | 0.0383 | 0.0087 |
| | | | | Sum | | 9.8985 | 34.6906 | 0.2853 | Down | | |
| | F3'5'H | K13083 (1.14.13.88) | 1599 | | | | | | | | |
| | DFR | K13082(1.1.1. | 1170 | | | | | | | | |

| | | | | | | | | | | | |
|---|---|---|---|---|---|---|---|---|---|---|---|
| | | 234/1.1.1.219 ) | | | | | | | | | |
| | ANS | K05277 （1.14.11.19） | 462 | | | | | | | | |
| | FLS | K05278 (1.14.20.6) | 1038 | Unigene2990 | 1267 | 119.9337 | 237.2475 | 0.5055 | Down | 0.1009 | 0.0283 |
| | | | | sum | | 119.9337 | 237.2475 | 0.5055 | Down | | |
| | ANR | K08695 （1.3.1.77） | 1041 | CL2486.Contig2 | 1423 | 21.3233 | 214.0709 | 0.0996 | Down | 0.0000 | 0.0000 |
| | | | | CL2486.Contig1 | 1834 | 2.8514 | 16.5003 | 0.1728 | Down | 0.2554 | 0.0927 |
| | | | | Sum | | 24.1747 | 230.5712 | 0.1048 | Down | | |
| | LAR | K13081 （1.17.1.3） | 1077 | CL54.Contig4 | 1634 | 0.8029 | 10.1991 | 0.0787 | Down | 0.1611 | 0.0508 |
| | | | | CL54.Contig3 | 1509 | 1.8325 | 221.8759 | 0.0083 | Down | 0.0000 | 0.0000 |
| | | | | CL54.Contig2 | 1396 | 0.9132 | 12.4012 | 0.0736 | Down | 0.1462 | 0.0449 |
| | | | | CL54.Contig1 | 1271 | 1.2597 | 4.6547 | 0.2706 | Down | 0.5422 | 0.2701 |
| | | | | Sum | | 4.8083 | 249.1308 | 0.0193 | Down | | |
| Transcription factors | MYB | K09422 | 738 | Unigene16444 | 2411 | 183.3128 | 748.0341 | 0.2451 | Down | 0.0000 | 0.0000 |
| | | | | Unigene14050 | 1143 | 19.2694 | 29.8014 | 0.6466 | Down | 0.4937 | 0.2335 |
| | | | | CL7.Contig2 | 2865 | 146.6169 | 0.3296 | 444.8723 | Up | 0.0000 | 0.0000 |
| | | | | Unigene16202 | 1048 | 13.6855 | 9.5435 | 1.4340 | Up | 0.7004 | 0.4846 |
| | | | | CL3418.Contig4 | 1381 | 0.2092 | 0.6780 | 0.3086 | Down | 0.6320 | 0.3541 |
| | | | | CL3418.Contig2 | 1341 | 86.4349 | 0.3112 | 277.7249 | Up | 0.0000 | 0.0000 |
| | | | | Unigene11816 | 1438 | 66.9351 | 97.7102 | 0.6850 | Down | 0.6829 | 0.4510 |
| | | | | Unigene5220 | 1183 | 17.2148 | 51.6077 | 0.3336 | Down | 0.0523 | 0.0127 |
| | | | | CL3418.Contig1 | 1530 | 2.0733 | 385.1637 | 0.0054 | Down | 0.0000 | 0.0000 |
| | | | | Unigene1252 | 1028 | 3.0084 | 12.0591 | 0.2495 | Down | 0.1403 | 0.0426 |
| | | | | Unigene17810 | 1302 | 29.2391 | 24.0746 | 1.2145 | Up | 0.7857 | 0.6122 |

| | | | | | | | | | | | |
|---|---|---|---|---|---|---|---|---|---|---|---|
| | | | | Unigene19345 | 1117 | 5.8893 | 37.4391 | 0.1573 | Down | 0.0101 | 0.0018 |
| | | | | Unigene14477 | 2650 | 184.2094 | 295.5953 | 0.6232 | Down | 0.2142 | 0.0735 |
| | | | | Sum | | 763.2877 | 1712.165 | 0.4458 | Down | | |
